# Fulminant Type 1 Diabetes as a Model of Nature to Explore the Role of C-Peptide

**DOI:** 10.1155/2008/819123

**Published:** 2008-04-15

**Authors:** Yuko Murase-Mishiba, A. Imagawa, Toshiaki Hanafusa

**Affiliations:** First Department of Internal Medicine, Osaka Medical College, 2-7 Daigaku-Machi, Takatsuki 569-8686, Japan

## Abstract

Patients with fulminant type 1 diabetes almost completely lack C-peptide even soon after the onset of the disease, and the deficiency continues for the rest of their life. Thus, fulminant type 1 diabetes could serve as a good model of nature to explore the physiological role of C-peptide. For example, patients with fulminant type 1 diabetes have diabetic chronic complications more frequently than those with classical autoimmune type 1 diabetes 5 years after the onset of diabetes, and the higher prevalence could be partly attributable to the complete lack of C-peptide in fulminant type 1 diabetes.

Type 1 diabetes mellitus is characterized by an insulin deficiency resulting from the destruction of pancreatic *β*-cells. Recently, a novel subtype of type 1 diabetes has been recorded and referred to as fulminant type 1 diabetes, which accounts for approximately 20% of Japanese ketosis-onset type 1 diabetes [1, 2]. Fulminant type 1 diabetes has the following clinical characteristics: duration of hyperglycemic symptoms is 4 days on average; a high prevalence of preceding common cold-like and gastrointestinal symptoms; a near-normal level of glycated hemoglobin in spite of very high plasma glucose levels associated with ketoacidosis, sometimes related to pregnancy; and increased serum pancreatic enzyme levels, absent C-peptide levels (fasting serum C-peptide < 0.10 nmol/L or stimulated serum C-peptide < 0.17 nmol/L soon after the disease onset), but virtually no detectable autoantibodies against constituents of pancreatic *β*-cells. The process of *β*-cell destruction is extremely rapid. Of note, in contrast to autoimmune type 1A diabetes, the deficiency of insulin secretory capacity has already become almost complete even at onset of diabetes, and the capacity rarely recovers after the onset. The patients are treated with recombinant human insulin, but C-peptide is not replaced. Thus, fulminant type 1 diabetes could serve as a good model of nature to explore the physiological role of C-peptide.

We conducted a nationwide survey in Japan to assess the development of microvascular complications in fulminant type 1 diabetes of 5 years’ duration in comparison with acute-onset autoimmune type 1A diabetes [3, 4]. Five-year cumulative incidence of microangiopathy was 24.4% in fulminant type 1 diabetes and 2.6% in type 1A diabetes. The cumulative incidence of each microangiopathy was significantly higher in fulminant type 1 diabetes than in type 1A diabetes; retinopathy was 9.8% versus 0% (*P* = .014), nephropathy 12.2% versus 2.6% (*P* = .015), and neuropathy 12.2% versus 1.3% (*P* = .010), respectively. Also, logistic regression analysis showed that decreased C-peptide secretion was a risk for retinopathy (*β* = 0.29; *P* = .04, *β* = −0.27; *P* < .05, resp.) and neuropathy (*β* = 0.39; *P* = .01, *β* = −0.25; *P* < .05, resp.). Mean HbA_1*c*_ levels were similar in fulminant and type 1A diabetes group during the follow-up periods. However, mean *M*-value, mean insulin dosages, and the frequency of severe hypoglycemic episodes were significantly higher, and mean postprandial C-peptide level was significantly lower in fulminant type 1 diabetes than in type 1A diabetes (0.08 ± 0.04 versus 0.24 ± 0.15 nmol/L, *P* = .0007). These results suggest that depleted and irreversible insulin production is associated with unstable blood glucose control, as indicated by increased *M*-value, and thereby high incidence of diabetic microvascular complications in fulminant type 1 diabetic patients. Here, the following interpretation is possible: lack of C-peptide itself (Figure 1), in addition to instability of glucose levels, might play a role in the development of microangiopathy in fulminant type 1 diabetic patients. Indeed, the mean postprandial C-peptide levels were almost undetectable even at the onset of diabetes (0.06 ± 0.03 nmol/L) and throughout the 5-year study in fulminant diabetic patients, while they were detectable at and decreased gradually after the onset in classical type 1A diabetes [5, 6].

C-peptide has been considered to be a good marker of insulin secretion and has no biological activity of its own. However, over the last decade, several reports have suggested that C-peptide exerts a number of physiological effects, which are probably mediated by stimulation of Na^+^, K^+^-ATPase, and endothelial nitric oxide synthetase activities in several tissues [7]. At the early stage of type 1 diabetes, C-peptide replacement was shown to result in diminished urinary albumin excretion rate and ameliorates nerve dysfunction [8]. In the light of these data, fulminant type 1 diabetes could provide an ideal setting to explore whether C-peptide administration benefits patients with diabetes in reducing microangiopathy.

## Figures and Tables

**Figure 1:**
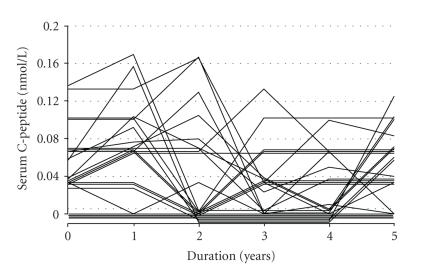
Serum C-peptide levels for 5 years from fulminant diabetes onset. Adapted from [4].
